# A population-based study on social inequality and barriers to healthcare-seeking with lung cancer symptoms

**DOI:** 10.1038/s41533-022-00314-7

**Published:** 2022-11-05

**Authors:** Lisa Maria Sele Sætre, Sanne Rasmussen, Kirubakaran Balasubramaniam, Jens Søndergaard, Dorte Ejg Jarbøl

**Affiliations:** Research Unit of General Practice, Department of Public Health, University of Southern Denmark, Odense, Denmark

**Keywords:** Respiratory signs and symptoms, Epidemiology

## Abstract

Healthcare-seeking with lung cancer symptoms is a prerequisite for improving timely diagnosis of lung cancer. In this study we aimed to explore barriers towards contacting the general practitioner (GP) with lung cancer symptoms, and to analyse the impact of social inequality. The study is based on a nationwide survey with 69,060 individuals aged ≥40 years, randomly selected from the Danish population. The survey included information on lung cancer symptoms, GP contacts, barriers to healthcare-seeking and smoking status. Information about socioeconomics was obtained by linkage to Danish Registers. Descriptive statistics and multivariate logistic regression model were used to analyse the data. “Being too busy” and “Being worried about wasting the doctor’s time” were the most frequent barriers to healthcare-seeking with lung cancer symptoms. Individuals out of workforce and individuals who smoked more often reported “Being worried about what the doctor might find” and “Being too embarrassed” about the symptoms. The social inequality in barriers to healthcare-seeking with lung cancer symptoms is noticeable, which emphasises the necessity of focus on vulnerable groups at risk of postponing relevant healthcare-seeking.

## Introduction

Lung cancer is one of the most common cancers and by far the most common cause of cancer related deaths worldwide^1,2^. The prognosis of cancer is highly dependent on the stage of the disease at diagnosis^3^. Partly due to great attention to timely diagnosis of cancer in recent years, the overall prognosis has improved significantly for several cancers in Denmark, but patients with lung cancer are still often diagnosed in an advanced stage^4,5^.

The physicians´ suspicion of a potential lung cancer diagnosis is often raised based on patient reported symptoms and supported by the patients’ smoking history^6^. A prerequisite for early diagnosis and improved survival rates is that patients contact their general practitioner (GP), when experiencing a symptom indicative of lung cancer^7^. The importance of seeking healthcare when experiencing symptoms has been emphasised to the general population by several cancer awareness campaigns, e.g. “The Seven Signs”, a nationwide campaign initiated by the Danish Cancer Society highlighting seven symptoms of different cancers through television adds, internet articles and other channels. A report evaluating the campaign concludes that the awareness for some cancer symptoms increased from 2017 to 2019 but emphasises the necessity of further efforts targeting healthcare-seeking with symptoms like coughing and difficulties swallowing and barriers to healthcare seeking^8^. However, healthcare-seeking is a complex process based upon social life and social network^9^, and among others affected by lifestyle factors and socioeconomic status^10–12^. Studies have shown that only about 40% of individuals from the general population with lung cancer symptoms report having contacted their GP about the symptoms, and that the proportion of GP contact is even lower among individuals who currently smoke where only one third report GP contact with lung cancer symptoms^11,13,14^.

Social inequality in lung cancer prevalence and mortality rates are persistent despite intentions towards the opposite^5^. The Danish Cancer Society estimates that most of the social inequality in cancer prevalence and mortality is due to smoking; however other factors, such as socioeconomic status should also be considered^15^. With smoking being the main risk factor for developing lung cancer, the ideal solution to reduce the social inequality in cancer survival would be to prevent future generations from initiating smoking. Given that previous smoking history cannot be changed, focus should also be on optimising lung cancer diagnostics among individuals who currently smoke or have formerly smoked. Screening for lung cancer among high risk groups have overall been found to increase the likelihood of early diagnosis of lung cancer and to decrease lung cancer mortality^16,17^, however the evidence also point to the fact that screening for lung cancer is not without challenges and derived side effects such as false positive results, psychosocial consequences^18,19^, and low participation rates among high risk group^17^. Lung cancer screening is currently being introduced and proposed as clinical trials in several countries, including Denmark^20,21^. However, not all lung cancers will be detected by screening, thus seeking healthcare when experiencing lung cancer symptoms is still of great importance^22^. Furthermore, understanding the healthcare-seeking behaviour among both individuals who currently smoke or have formerly smoked may be relevant regarding participation in the screening programs as well. Previous studies exploring social inequality in barriers to healthcare-seeking with lung cancer symptoms have mainly focused on socioeconomic status^4,23–25^, without considering smoking status. Thus, studies including both factors are of great importance to gain a more nuanced knowledge. This will be valuable in planning efforts focusing on both healthcare-seeking and awareness of lung cancer symptoms in the general population, as well as efforts to enhance the chance of the GPs’ recognising the symptoms as potential signs of cancer.

Qualitative studies have implied that individuals who currently smoke do not contact their GP due to e.g., fear of stigmatising, neglect, or normalisation of the symptoms^26,27^. Patients with lung cancer have described how they felt blame and shame when having to present lung related symptoms to their GP, and even more when receiving the cancer diagnosis^28^. Other patients with lung cancer describe how they feared receiving a smoking related diagnosis resulting in immediate healthcare-seeking for some and postponing healthcare-seeking for others^29^. Current knowledge about barriers to healthcare-seeking with lung cancer symptoms is based on research among lung cancer patients^30,31^ or on studies exploring anticipated barriers to healthcare-seeking^32^, which might be biased and difficult to apply to the understanding of the healthcare-seeking behaviour in the general population. Despite improvements in treatments and prognosis, more knowledge about barriers to healthcare-seeking with lung cancer symptoms is needed to improve early diagnosis and lung cancer survival rates.

Therefore, the objectives of this study were to explore the barriers to healthcare-seeking with lung cancer symptoms in the general population. Moreover, to explore the impact of social inequality by analysing the association between age, sex, smoking status and socioeconomic factors and barriers to healthcare-seeking.

## Results

### Study population

**Fig. 1:**
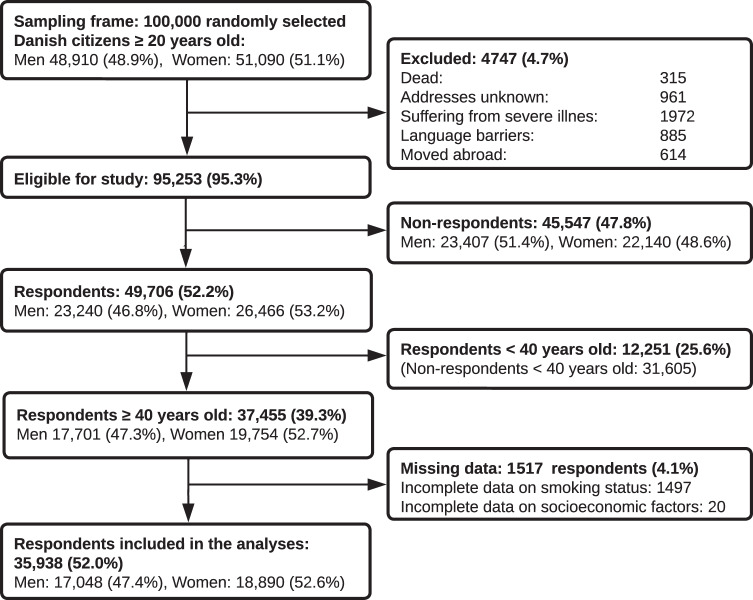
Flowchart. Flowchart of the study sample selection process.

Of the invited 100,000 randomly selected individuals, 4747 (4.7%) were not eligible because they had either died, could not be reached due to unknown address, were suffering from severe illness (including dementia), had language problems, or had moved abroad. Of the eligible 95,253 individuals, 49,706 completed the questionnaire, yielding an overall response rate of 52.2%. After exclusion of all individuals younger than 40 years old and exclusion of 1517 individuals with incomplete data due to missing this study includes a total of 35,938 respondents (52.0 %), Fig. 1.

More women (53.2%) than men (46.8%) 40 years or older answered the questionnaire. Some 20.9% of the respondents were individuals who currently smoked, and both individuals with low (14.0%) and high (33.0%) educational level participated. The majority of the respondents were married/living together (78.9%), and more than half were working (62.0%), while 4.1% was out of the workforce. Only a minority of the respondents were immigrants or descendants of immigrants (0.4%). The respondents were fairly representative of the study sample with regard to ethnicity, socioeconomic and demographic. A comparison of respondents and non-respondents is presented in detail elsewhere^10^.

The most frequent symptom was prolonged hoarseness (8.9%), while haemoptysis was the least frequent (0.1%). The proportion of contacts to the GP with lung cancer symptoms varied from 31.3% for prolonged hoarseness to 54.5% for shortness of breath, Table 1. Detailed multivariate analyses of the symptom experience and healthcare-seeking behaviour among individuals with lung cancer symptoms have been published elsewhere^11,33^. The proportion of GP contacts with each lung cancer symptom increased in frequency with 5–20% for individuals experiencing an additional specific lung cancer symptom. The increase in GP contacts were highest for prolonged coughing (11.9%) and haemoptysis (19.9%) and further accentuated by reporting current smoking. Adding report of a non-specific lung cancer symptom did not change the GP contact notably, except for haemoptysis, but the absolute numbers for haemoptysis are small. For all lung cancer symptoms, the proportion of GP contacts was highest in the oldest age group, among individuals who formerly smoked and among individuals with low educational level and individuals out of workforce, Table 1.

**Table 1:** Symptom prevalence and proportion of healthcare-seeking with single and combination of lung cancer symptoms (LCS).

		Prolonged coughing				Shortness of breath				Haemoptysis^a^				Prolonged hoarseness			
		As single symptom		+ Specific LCS	+ Specific and non-specific LCS	As single symptom		+ Specific LCS	+ Specific and non-specific LCS	As single symptom		+ Specific LCS	+ Specific and non-specific LCS	As single symptom		+ Specific LCS	+ Specific and non-specific LCS
	Total *N*	Symptom experience *n* (%)	Contact to GP *n* (%)	Contact to GP *n* (%)	Contact to GP *n* (%)	Symptom experience *n* (%)	Contact to GP *n* (%)	Contact to GP *n* (%)	Contact to GP *n* (%)	Symptom experience *n* (%)	Contact to GP *n* (%)	Contact to GP *n* (%)	Contact to GP *n* (%)	Symptom experience *n* (%)	Contact to GP *n* (%)	Contact to GP *n* (%)	Contact to GP *n* (%)
Total	35938	3215 (8.9)	1202 (37.4)	580 (49.3)	426 (48.7)	2898 (8.1)	1580 (54.5)	492 (57.1)	368 (55.3)	39 (0.1)	21 (53.8)	14 (73.7)	9 (90.0)	1323 (3.7)	414 (31.3)	254 (36.4)	195 (37.6)
Sex																	
Men	17048	1597 (9.4)	553 (34.6)	275 (48.7)	204 (48.3)	1449 (8.5)	806 (55.6)	245 (58.9)	184 (56.6)	24 (0.1)	13 (54.2)	–	–	655 (3.8)	194 (29.6)	112 (34.1)	81 (33.9)
Women	18890	1618 (8.6)	649 (40.1)	305 (49.9)	222 (49.0)	1449 (7.7)	774 (53.4)	247 (55.4)	184 (54.1)	15 (0.1)	8 (53.3)	–	–	668 (3.5)	220 (32.9)	142 (38.5)	114 (40.7)
Age-group																	
40–54 years	15565	1013 (6.5)	304 (30.0)	135 (41.2)	115 (41.5)	1083 (7.0)	461 (42.6)	117 (46.6)	99 (45.8)	15 (0.1)	7 (46.7)	–	–	364 (2.3)	94 (25.8)	59 (33.1)	56 (37.1)
55–69 years	14462	1476 (10.2)	554 (37.5)	260 (48.3)	186 (48.3)	1129 (7.8)	653 (57.8)	222 (57.4)	163 (56.4)	15 (0.1)	7 (46.7)	–	–	568 (3.9)	169 (29.8)	100 (33.0)	73 (33.3)
70- years	5911	726 (12.3)	344 (47.4)	185 (59.7)	125 (58.7)	686 (11.6)	466 (67.9)	153 (68.3)	106 (66.3)	9 (0.2)	7 (77.8)	–	–	391 (6.6)	151 (38.6)	95 (44.0)	66 (44.3)
Smoking status																	
Never smoking	15053	932 (6.2)	387 (41.5)	164 (49.2)	118 (48.4)	824 (5.5)	431 (52.3)	127 (54.7)	92 (52.3)	–	–	–	–	439 (2.9)	146 (33.3)	88 (41.9)	68 (42.8)
Former smoking	13384	1000 (7.5)	449 (44.9)	225 (54.7)	164 (53.6)	1231 (9.2)	748 (60.8)	192 (65.5)	143 (62.4)	–	–	–	–	572 (4.3)	190 (33.2)	116 (39.6)	84 (40.4)
Current smoking	7501	1283 (17.1)	366 (28.5)	191 (44.2)	144 (44.3)	843 (11.2)	401 (47.6)	173 (51.3)	133 (51.2)	–	–	–	–	312 (4.2)	78 (25.0)	50 (25.8)	43 (28.3)
Educational level																	
Low (<10 years)	5036	637 (8.8)	276 (43.3)	157 (54.1)	105 (51.7)	638 (12.7)	413 (64.7)	143 (61.4)	98 (59.8)	–	–	–	–	277 (5.5)	107 (38.6)	71 (43.3)	51 (43.2)
Middle (10–15 years)	19032	1683 (7.5)	626 (37.2)	296 (49.7)	231 (50.1)	1588 (8.3)	831 (52.3)	254 (58.0)	198 (56.6)	–	–	–	–	666 (3.5)	217 (32.6)	136 (38.9)	112 (41.8)
High (>= 15 years)	11870	895 (10.9)	300 (33.5)	127 (43.6)	90 (42.7)	672 (5.7)	336 (50.0)	95 (49.7)	72 (47.7)	–	–	–	–	380 (3.2)	90 (23.7)	47 (25.7)	32 (24.1)
Marital status																	
Single	7949	866 (10.9)	348 (40.2)	190 (54.1)	144 (53.1)	887 (11.2)	497 (56.0)	162 (61.8)	122 (58.9)	–	–	–	–	406 (5.1)	131 (32.3)	83 (36.6)	66 (37.1)
Married/living together	27989	2349 (8.4)	854 (36.4)	390 (47.3)	282 (46.7)	2011 (7.2)	1083 (53.9)	330 (55.0)	246 (53.7)	–	–	–	–	917 (3.3)	283 (30.9)	171 (36.4)	129 (37.8)
Labour market affiliation																	
Working	22285	1552 (7.0)	464 (29.9)	175 (38.0)	133 (37.9)	1315 (5.9)	583 (44.3)	145 (44.2)	113 (42.3)	–	–	–	–	552 2.5)	140 (25.4)	76 (30.0)	59 (31.9)
Pension	10646	1267 (11.9)	569 (44.9)	302 (58.0)	204 (57.1)	1103 (10.4)	729 (66.1)	252 (67.7)	171 (66.8)	–	–	–	–	613 (5.8)	218 (35.6)	135 (39.7)	96 (39.5)
Out of workforce	1469	147 (10.0)	61 (41.5)	31 (52.5)	29 (52.7)	163 (11.1)	86 (52.8)	31 (64.6)	29 (64.4)	–	–	–	–	51 (3.5)	15 (29.4)	10 (37.0)	10 (38.5)
Disability pension	1538	249 (16.2)	108 (43.4)	72 (52.9)	60 (53.6)	317 (20.6)	182 (57.4)	64 (56.1)	55 (56.7)	–	–	–	–	107 (7.0)	41 (38.3)	33 (42.9)	30 (46.2)
Ethnicity																	
Danish	34264	3054 (8.9)	1130 (37.0)	540 (48.7)	393 (48.2)	2730 (8.0)	1484 (54.4)	459 (56.7)	340 (55.0)	–	–	–	–	1265 (3.7)	394 (31.1)	238 (36.0)	182 (37.1)
Immigrants/descendants of immigrants	167	161 (9.6)	72 (44.7)	40 (58.8)	33 (55.0)	168 (10.0)	96 (57.1)	33 (63.5)	28 (59.6)	–	–	–	–	58 (3.5)	20 (34.5)	16 (45.7)	13 (46.4)

^a^Du to Danish legislation reporting of numbers ≤3 is not permitted.

### Barriers to healthcare-seeking with lung cancer symptoms

Table 2 shows the proportion of barriers to healthcare-seeking with each lung cancer symptom among individuals with no GP contact regarding their lung cancer symptom. Overall, between 38.2% and 47.7% of the respondents reported no barriers to healthcare-seeking. “Being too embarrassed” was the least frequent barrier reported by between 2.3% (prolonged hoarseness) and 3.2% (shortness of breath). For all the lung cancer symptoms almost one out of six reported “Being worried about wasting the doctor’s time” and “Being too busy”. The proportion of “Being worried about what the doctor might find” was highest for prolonged coughing (15.5%) and shortness of breath (15.1%), respectively, Table 2.

**Table 2:** Proportions of barriers to healthcare-seeking with lung cancer symptoms among individuals with no GP contact about their lung cancer symptoms.

	Prolonged coughing						Shortness of breath						Prolonged hoarseness					
	No GP-contact *n*	Too embar-rassing *n* (%)	Worried about wasting the doctor’s time *n* (%)	Worried about what the doctor might find *n* (%)	Too Busy *n* (%)	No barriers *n* (%)	No GP-contact *n*	Too embar-rassing *n* (%)	Worried about wasting The doctor’s time *n* (%)	Worried about what the doctor might find *n* (%)	Too Busy *n* (%)	No barriers *n* (%)	No GP-contact *n*	Too Embar-rassing *n* (%)	Worried about wasting the doctor’s time *n* (%)	Worried about what the doctor might find *n* (%)	Too Busy *n* (%)	No barriers *n* (%)
Total	2013	51 (2.5)	310 (15.4)	274 (13.6)	293 (14.6)	880 (43.7)	1318	42 (3.2)	192 (14.6)	206 (15.6)	221 (16.8)	503 (38.2)	909	21 (2.3)	150 (16.5)	80 (8.8)	137 (15.1)	434 (47.7)
Sex																		
Men	1044	23 (2.2)	147 (14.1)	129 (12.4)	162 (15.5)	499 (47.8)	643	20 (3.1)	86 (13.4)	109 (17.0)	118 (18.4)	261 (40.6)	461	13 (2.8)	78 (16.9)	36 (7.8)	77 (16.7)	233 (50.5)
Women	969	28 (2.9)	163 (16.8)	145 (15.0)	131 (13.5)	381 (39.3)	675	22 (3.3)	106 (15.7)	97 (14.4)	103 (15.3)	242 (35.9)	448	8 (1.8)	72 (16.1)	44 (9.8)	60 (13.4)	201 (44.9)
Age-group																		
40–54 years	709	24 (3.4)	124 (17.5)	119 (16.8)	163 (23.0)	242 (34.1)	622	25 (4.0)	115 (18.5)	123 (19.8)	157 (25.2)	179 (28.8)	270	7 (2.6)	56 (20.7)	30 (11.1)	69 (25.6)	91 (33.7)
55–69 years	922	22 (2.4)	151 (16.4)	122 (13.2)	111 (12.0)	418 (45.3)	476	12 (2.5)	49 (10.3)	64 (13.4)	53 (11.1)	205 (43.1)	399	9 (2.3)	66 (16.5)	32 (8.0)	51 (12.8)	200 (50.1)
70+ years	382	5 (1.3)	35 (9.2)	33 (8.6)	19 (5.0)	220 (57.6)	220	5 (2.3)	28 (12.7)	19 (8.6)	11 (5.0)	119 (54.1)	240	5 (2.1)	28 (11.7)	18 (7.5)	17 (7.1)	143 (59.6)
Smoking status																		
Never smoking	545	12 (2.2)	83 (15.2)	39 (7.2)	82 (15.0)	244 (44.8)	393	6 (1.5)	63 (16.0)	39 (9.9)	72 (18.3)	144 (36.6)	293	–	53 (18.1)	24 (8.2)	52 (17.7)	121 (41.3)
Former smoking	551	4 (0.7)	61 (11.1)	46 (8.3)	69 (12.5)	272 (49.4)	483	7 (1.4)	55 (11.4)	48 (9.9)	66 (13.7)	197 (40.8)	382	–	54 (14.1)	27 (7.1)	47 (12.3)	202 (52.9)
Current smoking	917	35 (3.8)	166 (18.1)	189 (20.6)	142 (15.5)	364 (39.7)	442	29 (6.6)	74 (16.7)	119 (26.9)	83 (18.8)	162 (36.7)	234	–	43 (18.4)	29 (12.4)	38 (16.2)	111 (47.4)
Educational level																		
Low (<10 years)	361	11 (3.0)	48 (13.3)	60 (16.6)	39 (10.8)	183 (50.7)	225	5 (2.2)	34 (15.1)	41 (18.2)	23 (10.2)	113 (50.2)	170	–	21 (12.4)	13 (7.6)	14 (8.2)	107 (62.9)
Middle (10–14 years)	1057	28 (2.6)	177 (16.7)	135 (12.8)	162 (15.3)	475 (44.9)	757	25 (3.3)	112 (14.8)	122 (16.1)	136 (18.0)	295 (39.0)	449	–	80 (17.8)	46 (10.2)	68 (15.1)	219 (48.8)
High (≥15 years)	595	12 (2.0)	85 (14.3)	79 (13.3)	92 (15.5)	222 (37.3)	336	12 (3.6)	46 (13.7)	43 (12.8)	62 (18.5)	95 (28.3)	290	–	49 (16.9)	21 (7.2)	55 (19.0)	108 (37.2)
Marital Status																		
Single	518	13 (2.5)	81 (15.6)	80 (15.4)	71 (13.7)	217 (41.9)	390	16 (4.1)	59 (15.1)	66 (16.9)	57 (14.6)	143 (36.7)	275	6 (2.2)	44 (16.0)	24 (8.7)	44 (16.0)	133 (48.4)
Married/living together	1495	38 (2.5)	229 (15.3)	194 (13.0)	222 (14.8)	663 (44.3)	928	26 (2.8)	133 (14.3)	140 (15.1)	164 (17.7)	360 (38.8)	634	15 (2.4)	106 (16.7)	56 (8.8)	93 (14.7)	301 (47.5)
Labour Market affiliation																		
Working	1088	22 (2.0)	163 (15.0)	165 (15.2)	230 (21.1)	411 (37.8)	732	22 (3.0)	119 (16.3)	130 (17.8)	183 (25.0)	223 (30.5)	412	7 (1.7)	76 (18.4)	–	–	159 (38.6)
Pension	698	13 (1.9)	100 (14.3)	66 (9.5)	43 (6.2)	368 (52.7)	374	10 (2.7)	39 (10.4)	35 (9.4)	21 (5.6)	194 (51.9)	395	7 (1.8)	55 (13.9)	–	–	225 (57.0)
Out of workforce	86	7 (8.1)	22 (25.6)	18 (20.9)	8 (9.3)	29 (33.7)	77	4 (4.7)	15 (19.5)	13 (16.9)	8 (10.4)	27 (35.1)	36	≤3^a^	11 (30.6)	–	–	13 (36.1)
Disability pension	141	9 (6.4)	25 (17.7)	25 (17.7)	12 (8.5)	72 (51.1)	135	6 (4.4)	19 (14.1)	28 (20.7)	9 (6.7)	59 (43.7)	66	≤3^a^	8 (12.1)	–	–	37 (56.1)
Ethnicity																		
Danish	1924	43 (2.2)	292 (15.2)	258 (13.4)	274 (14.2)	849 (44.1)	1246	37 (3.0)	174 (14.0)	192 (15.4)	203 (16.3)	478 (38.4)	871	–	136 (15.6)	–	124 (14.2)	420 (48.2)
Immigrants/descendants of immigrants	89	8 (9.0)	18 (20.2)	16 (18.0)	19 (21.3)	31 (34.8)	72	5 (6.9)	18 (25.0)	14 (19.4)	18 (25.0)	25 (34.7)	38	–	14 (36.8)	–	13 (34.2)	14 (36.8)

^a^Due to Danish legislation reporting of numbers ≤3 is not permitted.

### Social inequality and barriers to healthcare-seeking with lung cancer symptoms

The associations between barriers to healthcare-seeking and each covariate are shown in Table 3A–C with both crude and adjusted odds ratios (OR) and 95% Confidence Intervals (CI). Individuals in the oldest age group had significantly lower odds of “Being worried about wasting the doctor’s time” (Adj. OR 0.48, 95% CI: 0.32–0.71) for prolonged coughing and of “Being too busy” for all symptoms, compared to the youngest age group. Individuals who currently smoked were three times more likely to report “Being worried about what the doctor might find” for both prolonged coughing (Adj. OR 3.26, 95% CI: 2.26–4.69) and shortness of breath (Adj. OR: 3.40, 95% CI: 2.28–5.06), compared to individuals who had never smoked. Furthermore, individuals who currently smoked reporting shortness of breath were almost five times more likely to report “Being too embarrassed” (Adj. OR 4.73, 95% CI: 1.93–11.61) compared to the individuals who never smoked. Further, immigrants or descendants of immigrants with prolonged coughing had higher odds of reporting “Being too embarrassed” (Adj. OR 3.48, 95% CI: 1.49–8.13) compared to individuals with Danish ethnicity. The same tendency was seen for shortness of breath, however not statistically significant. For prolonged coughing and shortness of breath, individuals out of workforce (Adj. OR_prolonged coughing_ 0.33 95% CI: 0.15–0.69, Adj. OR_Shortness of breath_ 0.29 95% CI: 0.13–0.62) were less than half as likely to report “Being too busy” compared to individuals working, Table 3A, B. The same was found for individuals on pension (Adj. OR_prolonged coughing_ 0.43 95% CI: 0.27–0.68, Adj. OR_Shortness of breath_ 0.37, 95% CI: 0.19–0.72) and individuals on disability pension (Adj. OR_prolonged coughing_ 0.43 95% CI: 0.27–0.68, Adj. OR_Shortness of breath_ 0.23, 95% CI: 0.12–0.47).

**Table 3:** A: Associations between sex, age, smoking status, socioeconomic status, and barriers to contact to GP with prolonged coughing. B: Associations between sex, age, smoking status, socioeconomic status, and barriers to contact to GP with shortness of breath. C: Associations between sex, age, smoking status, socioeconomic status, and barriers to contact to GP with prolonged hoarseness.

Prolonged coughing										
	Too embarrassing		Worried about wasting the doctor’s time		Worried about what the doctor might find		Too busy		No barriers	
	OR_crude_ (95% CI)	OR_Adj._^a^ (95% CI)	OR_crude_ (95% CI)	OR_Adj._^a^ (95% CI)	OR_crude_ (95% CI)	OR_Adj._^a^ (95% CI)	OR_crude_ (95% CI)	OR_Adj._^a^ (95% CI)	OR_crude_ (95% CI)	OR_Adj._^a^ (95% CI)
Sex										
Men	1	1	1	1	1	1	1	1	1	1
Women	1.32 (0.76–2.31)	1.24 (0.70–2.19)	1.23 (0.97–1.57)	1.20 (0.94–1.54)	1.25 (0.97–1.61)	**1.33 (1.02–1.73)**	0.85 (0.66–1.09)	0.87 (0.67–1.12)	**0.71 (0.59–0.84)**	**0.69 (0.57–0.82)**
Age										
40–54 years	1	1	1	1	1	1	1	1	1	1
55–69 years	0.70 (0.39–1.25)	0.73 (0.37–1.47)	0.92 (0.71–1.20)	0.83 (0.61–1.13)	0.76 (0.57–0.99)	0.89 (0.65–1.22)	**0.46 (0.35–0.60)**	**0.58 (0.43–0.78)**	**1.60 (1.31–1.96)**	**1.46 (1.16–1.83)**
70+ years	0.38 (0.14–1.00)	0.46 (0.12–1.70)	**0.48 (0.32–0.71)**	**0.36 (0.21–0.61)**	**0.47 (0.31–0.70)**	0.80 (0.45–1.44)	**0.18 (0.11–0.29)**	**0.35 (0.18–0.68)**	**2.62 (2.03–3.38)**	**2.17 (1.50–3.14)**
Smoking status										
Never smoking	1	1	1	1	1	1	1	1	1	1
Former smoking	0.32 (0.10–1.01)	0.37 (0.12–1.16)	0.69 (0.49–0.99)	0.74 (0.52–1.06)	1.18 (0.76–1.84)	1.28 (0.82–2.01)	0.81 (0.57–1.14)	1.05 (0.73–1.50)	1.20 (0.95–1.53)	1.04 (0.81–1.33)
Current smoking	1.76 (0.91–3.43)	1.65 (0.83–3.25)	1.23 (0.92–1.64)	1.19 (0.89–1.60)	**3.37 (2.34–4.84)**	**3.26 (2.26–4.69)**	1.03 (0.77–1.39)	1.01 (0.74–1.37)	0.81 (0.66–1.01)	0.82 (0.66–1.03)
Educational level										
Low (<10 years)	1	1	1	1	1	1	1	1	1	1
Middle (10–14 years)	0.87 (0.43–1.76)	0.81 (0.39–1.70)	1.31 (0.93–1.85)	1.28 (0.89–1.82)	0.73 (0.53–1.02)	0.66 (0.47–0.94)	**1.49 (1.03–2.17)**	0.99 (0.67–1.47)	0.79 (0.62–1.01)	0.95 (0.74–1.21)
High (≥15 years)	0.65 (0.29–1.50)	0.68 (0.28–1.63)	1.09 (0.74–1.59)	1.10 (0.74–1.64)	0.77 (0.53–1.11)	0.79 (0.53–1.16)	**1.51 (1.01–2.25)**	0.94 (0.62–1.44)	**0.58 (0.44–0.75)**	**0.68 (0.52–0.90)**
Marital status										
Single	1	1	1	1	1	1	1	1	1	1
Married/living together	1.01 (0.54–1.92)	1.23 (0.64–2.38)	0.98 (0.74–1.29)	1.00 (0.75–1.33)	0.82 (0.62–1.08)	0.89 (0.67–1.19)	1.10 (0.82–1.46)	0.97 (0.72–1.31)	1.11 (0.90–1.35)	1.17 (0.95–1.44)
Labour Market affiliation										
Working	1	1	1	1	1	1	1	1	1	1
Pension	0.92 (0.46–1.84)	1.71 (0.67–4.34)	0.95 (0.72–1.24)	1.66 (1.15–2.38)	0.58 (0.43–0.79)	0.76 (0.50–1.16)	**0.24 (0.17–0.34)**	**0.43 (0.27–0.68)**	1.84 (1.52–2.23)	1.17 (0.89–1.54)
Out of workforce	**4.29 (1.78–10.36)**	**2.79 (1.10–7.09)**	**1.95 (1.17–3.26)**	**1.76 (1.04–2.97)**	1.48 (0.86–2.55)	1.24 (0.70–2.18)	**0.38 (0.18–0.80)**	**0.33 (0.15–0.69)**	0.84 (0.53–1.33)	0.95 (0.59–1.52)
Disability pension	**3.30 (1.49–7.33)**	**3.17 (1.40–7.18)**	1.22 (0.77–1.94)	1.20 (0.75–1.93)	1.21 (0.76–1.92)	1.03 (0.64–1.66)	**0.35 (0.19–0.64)**	**0.38 (0.20–0.69)**	**1.72 (1.21–2.44)**	**1.69 (1.18–2.42)**
Ethnicity										
Danish	1	1	1	1	1	1	1	1	1	1
Immigrants/descendants of immigrants	**4.32 (1.97–9.49)**	**3.48 (1.49–8.13)**	1.42 (0.83–2.41)	1.24 (0.71–2.15)	1.42 (0.81–2.47)	1.34 (0.75–2.40)	1.63 (0.97–2.76)	1.62 (0.94–2.81)	0.68 (0.43–1.06)	0.76 (0.48–1.20)

Shortness of breath										
	Too embarrassing		Worried about wasting the doctor’s time		Worried about what the doctor might find		Too busy		No barriers	
	OR_crude_ (95% CI)	OR_Adj._^a^ (95% CI)	OR_crude_ (95% CI)	OR_Adj._^a^ (95% CI)	OR_crude_ (95% CI)	OR_Adj._^a^ (95% CI)	OR_crude_ (95% CI)	OR_Adj._^a^ (95% CI)	OR_crude_ (95% CI)	OR_Adj._^a^ (95% CI)
Sex										
Men	1	1	1	1	1	1	1	1	1	1
Women	1.05 (0.57–1.94)	1.14 (0.61–2.15)	1.21 (0.89–1.64)	1.18 (0.86–1.61)	0.82 (0.61–1.11)	0.86 (0.63–1.17)	0.80 (0.60–1.07)	0.80 (0.59–1.09)	0.82 (0.65–1.02)	0.80 (0.64–1.01)
Age										
40–54 years	1	1	1	1	1	1	1	1	1	1
55–69 years	0.62 (0.31–1.24)	0.55 (0.23–1.32)	**0.51 (0.35–0.72)**	**0.63 (0.42–0.95)**	**0.63 (0.45–0.88)**	0.68 (0.46–1.00)	**0.37 (0.26–0.52)**	**0.50 (0.34–0.73)**	**1.87 (1.46–2.41)**	**1.60 (1.20–2.15)**
70+ years	0.56 (0.21–1.47)	0.44 (0.10–1.99)	0.64 (0.41–1.00)	1.14 (0.53–2.45)	**0.38 (0.23–0.64)**	0.59 (0.27–1.26)	**0.16 (0.08–0.29)**	**0.35 (0.14–0.86)**	**2.92 (2.12–4.00)**	**2.04 (1.25–3.32)**
Smoking status										
Never smoking	1	1	1	1	1	1	1	1	1	1
Former smoking	0.95 (0.32–2.85)	1.08 (0.35–3.28)	0.67 (0.46–0.99)	0.78 (0.52–1.16)	1.00 (0.64–1.56)	1.15 (0.73–1.82)	0.71 (0.49–1.02)	0.97 (0.66–1.43)	1.19 (0.91–1.57)	0.98 (0.73–1.30)
Current smoking	**4.53 (1.86–11.03)**	**4.73 (1.93–11.61)**	1.05 (0.73–1.52)	1.09 (0.75–1.58)	**3.34 (2.26–4.95)**	**3.40 (2.28–5.06)**	1.03 (0.73–1.46)	1.16 (0.81–1.68)	1.00 (0.75–1.33)	0.98 (0.73–1.31)
Educational level										
Low (<10 years)	1	1	1	1	1	1	1	1	1	1
Middle (10–14 years)	1.50 (0.57–3.97)	1.51 (0.55–4.13)	0.98 (0.64–1.48)	0.78 (0.50–1.22)	0.86 (0.58–1.27)	0.73 (0.48–1.12)	**1.92 (1.20–3.08)**	1.11 (0.67–1.84)	**0.63 (0.47–0.85)**	0.82 (0.60–1.13)
High (≥15 years)	1.63 (0.57–4.69)	1.93 (0.64–5.80)	0.89 (0.55–1.44)	0.69 (0.42–1.15)	0.66 (0.41–1.05)	0.61 (0.37–1.02)	**1.99 (1.19–3.32)**	1.09 (0.63–1.89)	**0.39 (0.27–0.56)**	**0.51 (0.35–0.74)**
Marital status										
Single	1	1	1	1	1	1	1	1	1	1
Married/living together	0.67 (0.36–1.27)	0.79 (0.41–1.53)	0.94 (0.67–1.31)	0.95 (0.68–1.34)	0.87 (0.63–1.20)	0.96 (0.68–1.34)	1.25 (0.90–1.74)	1.09 (0.77–1.53)	1.09 (0.86–1.40)	1.20 (0.93–1.54)
Labour Market affiliation										
Working	1	1	1	1	1	1	1	1	1	1
Pension	0.89 (0.42–1.89)	1.95 (0.59–6.43)	**0.60 (0.41–0.88)**	0.62 (0.33–1.20)	**0.48 (0.32–0.71)**	0.76 (0.42–1.39)	**0.18 (0.11–0.29)**	**0.37 (0.19–0.72)**	**2.46 (1.90–3.18)**	**1.58 (1.08–2.31)**
Out of workforce	1.77 (0.59–5.27)	1.19 (0.39–3.68)	1.25 (0.69–2.26)	1.05 (0.57–1.94)	0.94 (0.50–1.76)	0.73 (0.38–1.40)	**0.35 (0.16–0.74)**	**0.29 (0.13–0.62)**	1.23 (0.75–2.02)	1.32 (0.80–2.18)
Disability pension	1.50 (0.60–3.77)	1.38 (0.53–3.57)	0.84 (0.50–1.42)	0.91 (0.53–1.56)	1.21 (0.77–1.91)	1.11 (0.69–1.79)	**0.21 (0.11–0.43)**	**0.23 (0.12–0.47)**	**1.77 (1.22–2.58)**	**1.62 (1.10–2.38)**
Ethnicity										
Danish	1	1	1	1	1	1	1	1	1	1
Immigrants/descendants of immigrants	2.44 (0.93–6.41)	2.15 (0.78–5.91)	**2.05 (1.18–3.58)**	1.73 (0.98–3.07)	1.33 (0.72–2.42)	1.20 (0.63–2.26)	1.71 (0.98–2.98)	1.57 (0.87–2.83)	0.85 (0.52–1.41)	1.01 (0.60–1.69)

Prolonged hoarseness										
	Too embarrassing		Worried about wasting the doctor’s time		Worried about what the doctor might find		Too busy		No barriers	
	OR_crude_ (95% CI)	OR_Adj_^a^ (95% CI)	OR_crude_ (95% CI)	OR_Adj._^a^ (95% CI)	OR_crude_ (95% CI)	OR_Adj._^a^ (95% CI)	OR_crude_ (95% CI)	OR_Adj._^a^ (95% CI)	OR_crude_ (95% CI)	OR_Adj._^a^ (95% CI)
Sex										
Men	1	–	1	1	1	1	1	1	1	1
Women	0.63 (0.26–1.53)	–	0.88 (0.61–1.26)	1.29 (0.81–2.04)	1.23 (0.77–1.97)	0.77 (0.53–1.11)	0.70 (0.47–1.03)	0.80 (0.61–1.03)	0.85 (0.64–1.11)	0.88 (0.61–1.26)
Age										
40–54 years	1	–	1	1	1	1	1	1	1	1
55–69 years	0.87 (0.32–2.36)	–	0.76 (0.51–1.12)	0.79 (0.50–1.25)	0.70 (0.41–1.18)	0.72 (0.39–1.31)	**0.43 (0.29–0.64)**	**0.61 (0.39–0.96)**	**1.98 (1.44–2.72)**	**1.75 (1.22–2.51)**
70+ years	0.80 (0.25–2.55)	–	**0.50 (0.31–0.83)**	0.54 (0.26–1.12)	0.65 (0.35–1.20)	0.76 (0.29–2.01)	**0.22 (0.13–0.39)**	0.67 (0.27–1.61)	**2.90 (2.02–4.16)**	**2.26 (1.31–3.90)**
Smoking status										
Never smoking	–	–	1	1	1	1	1	1	1	1
Former smoking	–	–	0.75 (0.49–1.13)	0.82 (0.54–1.25)	0.85 (0.48–1.51)	0.86 (0.48–1.55)	0.65 (0.42–1.00)	0.77 (0.49–1.21)	**1.60 (1.17–2.17)**	**1.40 (1.02–1.93)**
Current smoking	–	–	1.02 (0.65–1.59)	0.99 (0.63–1.57)	1.59 (0.90–2.81)	1.51 (0.84–2.70)	0.90 (0.57–1.42)	0.82 (0.51–1.33)	1.28 (0.91–1.81)	1.42 (0.99–2.04)
Educational level										
Low (<10 years)	–	–	1	1	1	1	1	1	1	1
Middle (10–14 years)	–	–	1.54 (0.92–2.58)	1.35 (0.79–2.30)	1.38 (0.72–2.62)	1.37 (0.70–2.66)	**1.99 (1.09–3.64)**	1.36 (0.72–2.59)	**0.56 (0.39–0.81**)	0.69 (0.47–1.00)
High (≥15 years)	–	–	1.44 (0.83–2.50)	1.17 (0.66–2.09)	0.94 (0.46–1.94)	0.93 (0.44–1.99)	**2.61 (1.40–4.85)**	1.51 (0.77–2.95)	**0.35 (0.24–0.52)**	**0.47 (0.31–0.72)**
Marital status										
Single	–	–	1	1	1	1	1	1	1	1
Married/living together	–	–	1.05 (0.72–1.55)	0.99 (0.67–1.48)	1.01 (0.61–1.67)	1.05 (0.63–1.77)	0.90 (0.61–1.33)	0.69 (0.45–1.05)	0.97 (0.73–1.28)	1.12 (0.83–1.51)
Labour Market affiliation										
Working	–	–	1	1	–	–	–	–	1	1
Pension	–	–	0.72 (0.49–1.04)	1.06 (0.61–1.82)	–	–	–	–	**2.11 (1.59–2.79)**	1.35 (0.90–2.03)
Out of workforce	–	–	1.95 (0.92–4.12)	1.67 (0.77–3.62)	–	–	–	–	0.90 (0.44–1.83)	0.97 (0.47–2.01)
Disability pension	–	–	0.61 (0.28–1.33)	0.59 (0.27–1.31)	–	–	–	–	**2.03 (1.20–3.43)**	**1.89 (1.10–3.22)**
Ethnicity										
Danish	–	–	1	1	–	–	1	1	1	1
Immigrants/descendants of immigrants	–	–	**3.15 (1.59–6.25)**	**2.86 (1.42–5.75)**	–	–	**3.13 (1.56–6.29)**	**3.24 (1.53–6.84)**	0.63 (0.32–1.23)	0.71 (0.36–1.42)

Bold indicates *P*-value < 0.05.

^a^Adj.= Adjusted for possible confounders: age, smoking status, labour market affiliation, educational level, and ethnicity.

## Discussion

This study adds to the symptom and population-based knowledge about healthcare-seeking behaviour and barriers to healthcare-seeking among individuals reporting lung cancer symptoms in the general population. In the present study a new aspect of interest was the influence of reporting more than one lung cancer symptom on the healthcare-seeking behaviour. We found that experience of two specific lung cancer symptoms increased the proportions who sought healthcare, while an additional non-specific symptom did not change the healthcare-seeking behaviour. Further, this study points to groups of individuals, e.g., individuals who currently smoke, immigrants and individuals out of workforce, who report more barriers towards contacting their GP regarding lung cancer symptoms and thereby are at risk of postponing relevant healthcare-seeking. Overall, healthcare-seeking with lung cancer symptoms varied from 31.3% (prolonged hoarseness) to 54.5% (shortness of breath). Among individuals with no GP contact, almost two out of three reported at least one barrier to healthcare-seeking. The most frequent reported barriers were “Being worried about what the doctor might find” and “Being too busy”, while reporting “Being too embarrassed” was rare. Social inequalities were noticeable for several of the barriers to healthcare-seeking with lung cancer symptoms. For prolonged coughing and shortness of breath individuals who currently smoked were three times more likely to report “Being worried about what the doctor might find” than individuals who never smoked, and five times more likely to report “Being too embarrassed” about shortness of breath. Moreover, individuals not working and immigrants or descendants of immigrants with prolonged coughing were more likely to report “Being too embarrassed“ and individuals out of workforce were more likely to report “Being worried about what the doctor might find”.

A strength of this study is the large sample of individuals from the general Danish population. The response rate of 52.0% among individuals older than 40 years old is higher than in previous surveys exploring symptoms and healthcare-seeking in the general population^13,34^. Although more respondents were women and the respondents were slightly older with higher income than the non-respondents, the respondents were fairly representative of the general Danish population^10^. Despite the large sample some subgroups, such as individuals who formerly smoked and immigrants or descendants of immigrants, are small, which make some of the results less certain.

Another strength of the study is the comprehensive work which was made prior to development of the questionnaire. The conceptual framework for the questionnaire and definitions of essential constructs as symptom experience and healthcare-seeking behaviour was discussed, evaluated, and defined by a multidisciplinary group. The conceptual framework was based on both theoretically and clinically based knowledge as well as existing literature^35^. Furthermore, both pilot-, and field testing of the study was made prior to distribution to assure comprehensibility and acceptability of each item and the questionnaire in its entirety, the details are described in Rasmussen et al.^36^.

Individuals with several symptoms or GP contacts may be more prone to participate in a survey concerning symptoms and healthcare-seeking, which could give rise to a risk of selection bias and induce a slight overestimation of the symptom prevalence and healthcare-seeking. On the other hand, individuals with many symptoms or GP contacts might not have the surplus of energy to answer a comprehensive questionnaire, which could counterbalance the estimate. The participants were asked about experience of lung cancer symptoms within the preceding 4 weeks, and whether they had contacted their GP about the symptoms. Individuals with no GP contact were asked about barriers to healthcare-seeking. Even though it seems reasonable to assume that symptom experiences can be accurately recalled within a 4-week time span recall bias cannot be eliminated. This might slightly underestimate the symptom prevalence, overestimate the GP contacts, and underestimate the proportion of barriers. The most significant symptoms might be easier to recall, and at the same time they will be the ones leading to most GP contacts and fewest barriers to healthcare-seeking. The fact that the survey was web-based may have caused especially elderly people not to participate, thus a telephone interview was offered instead, to reduce the selection bias. Data was collected in 2012, however the results are still relevant. The overall healthcare-seeking behaviour might have changed somewhat, but neither the social inequality in the healthcare-system or stigmatising of individuals who smoke, and smoking-related diagnoses have been improved^37–39^. Thus, results of this study will add to efforts targeting more equal access to and communication with both the GPs and the overall healthcare system.

The participants had the opportunity to describe other barriers to healthcare-seeking with lung cancer symptoms in a textbox. A profound qualitative data analysis of the comments is beyond the scope of this article, but overall, the comments included the following statements: “*already knowing the cause of the symptoms e.g., individuals who currently smoke or individuals having a chronic respiratory disease, not wishing to talk to the GP about smoking related subjects, and lack of trust to the GP*”. These comments are in line with both the qualitative literature, and a British study exploring organisational and relational barriers to healthcare-seeking with colorectal cancer symptoms^40^. Future population-based studies should explore both organisational and relational barriers to healthcare-seeking as well.

The proportion of barriers to healthcare-seeking with lung cancer symptoms found in this study were similar to the ones reported by Jarbøl et al. for healthcare-seeking with symptoms of colorectal cancer in the general population^41^ and by Balasubramaniam et al. for healthcare-seeking with gynaecological cancer symptoms in the general population^42^. Compared to the present study, Hvidberg et al. found higher estimates of barriers to healthcare-seeking^43^. The differences might be explained by the fact that Hvidberg et al. explored anticipated barriers to seeking healthcare, while we explored the reported barriers when experiencing symptoms. Similarly, to this study Hvidberg et al. also found decreasing odds of reporting barriers with increasing age. Also using barriers from the ABC measures Forbes et al. compared the beliefs about barriers to healthcare-seeking across several countries and found that Danish citizens have less anticipated barriers to symptomatic presentation than citizens in Norway, Sweden, Canada, United Kingdom (UK) and Australia^32^. Based on the same data Donnelly et al. reported results showing that high barrier score was associated with increased anticipated time to healthcare-seeking with e.g., coughing^44^. Even though a direct comparison is not possible, we find similar tendencies in the present study, with more barriers reported among individuals who currently smoke, who also have low likelihood of seeking healthcare^11,13^.

In contrast to this study and three other studies exploring healthcare-seeking in the general population^11,13,14^, a recently published review by van Os et al. concluded that individuals who never smoked are less likely to perceive lung cancer symptoms as signs of disease enhancing the time of symptom appraisal and postponing healthcare-seeking compared to individuals who currently smoke^45^. The review is, however, solely based on studies with individuals already diagnosed with lung cancer inducing risk of information bias which might reduce the representative of the behaviour in the general population.

We found that healthcare-seeking was higher among individuals with combinations of lung cancer symptoms. The difference was largest among individuals who currently smoked with prolonged coughing. This may be explained by coughing being a part of everyday life for many individuals who currently smoke^33^, thus not inciting healthcare-seeking, until another symptom occurs and triggers the awareness. In the update of the Danish lung cancer guideline in 2018 “change in a known cough” was added as a symptom of lung cancer. This might emphasize, that in individuals for whom coughing is a part of everyday life, even small changes in frequency, intensity or sputum should raise suspicion of lung cancer. How the general population understand the symptom “change in a known cough”, and whether it has been well implemented in clinical practice is unknown and should be explored in future studies.

The proportion of individuals reporting “Being too embarrassed” was rather low in the current study, implying that lung cancer symptoms, in general, are not embarrassing. However, individuals who currently smoked were five times more likely to report embarrassment about shortness of breath than individuals who never smoked. This is consistent with the qualitative literature on stigma and blame regarding smoking, lung cancer symptoms and diagnosis^26,29,30,38^. Likewise, literature exploring stigma in regard to smoking and chronic obstructive pulmonary disease have found that some individuals who smoke are very embarrassed and feel guilty about their symptoms, which might lead to omitting healthcare-seeking^39^. Further, individuals who currently smoked were more likely to report “Being worried about what the doctor might find”, which is illustrated by the statements in the study by Saab et al.*: “Seeking healthcare means being ill and being ill means death*”^46^. Another group who was also embarrassed about the symptoms were individuals with another ethnicity than Danish. Scheppers et al. published a review of potential barriers to healthcare-seeking among ethnic minorities and did not find embarrassment or shame to be an ethnicity-related barriers. However, they accentuated several other barriers such as language skills and perceived cause of the symptom or illness as ethnicity-related barriers, which when individuals is asked to tick off predefined barriers might be interpreted as a kind of embarrassment^47^.

One of six individuals who currently smoked reported “Being worried about wasting the doctor’s time”, which is almost the same frequency as among individuals who never smoked. For physicians, the combination of lung cancer symptoms and smoking is alarming, but individuals who currently smoke do not necessarily interpretate the symptoms as signs of potential severe illness^48^. Moreover, they may feel guilty and expect that the symptoms are self-inflicted by smoking leading to postponed or omitted healthcare-seeking^30^.

In this study we found that individuals out of workforce were more likely to report “Being too embarrassed” and “Being worried about what the doctor might find”. Further we found that the oldest age groups and individuals out of workforce, on pension and disability pension were less likely to report “Being too busy”. This is in line with some of the findings in the review by McCutchan et al. reviewing barriers to healthcare-seeking with symptoms of different cancer types^25^. McCutchan et al. found that individuals with lower socioeconomic status report more emotional barriers, such as being worried or embarrassed, while individuals with higher socioeconomic status and younger age groups report more practical barriers, such as being too busy. Moreover, McCuthan et al. also describe some other associations between socioeconomic status and barriers to healthcare-seeking, which were not found in the current study^25^. This discrepancy may be explained by the inclusion of smoking status in our analyses. In the crude analyses we found some statistically significant associations between the barriers and other socioeconomic factors, but after adjusting for smoking status, the associations were not significant. This concur with the estimation from the Danish Cancer Society, that most of the social inequality in cancer is due to smoking^49^. Thus, future interventions in addition to awareness of symptoms could target barriers to healthcare-seeking, especially among the groups experiencing the most barriers. For instance, individuals out of workforce, might need more support already before they contact the GP. This could be through guidance from social workers or at the public employment centre^50^. Further, if GPs verbalise barriers to healthcare-seeking when having consultations with e.g., individuals who smoke and immigrants or descendants of immigrants, it may decrease the barriers to healthcare-seeking henceforth.

## Conclusion

Healthcare-seeking with lung cancer symptoms varied from 37.5% to 53.5% and increased when additional lung cancer symptoms were reported. Almost two out of three individuals with no GP contact regarding their lung cancer symptoms reported one of the predefined barriers to healthcare-seeking. “Being worried about wasting the doctor’s time” and “Being too busy” were the most frequent reported barriers for all lung cancer symptoms. For prolonged coughing and shortness of breath “Being worried about what the doctor might find” was frequent as well.

Social inequality was reflected by individuals who currently smoked with prolonged coughing and shortness of breath being three times more likely to report “Being worried about what the doctor might find”, and individuals who currently smoked with shortness of breath being five times more likely to report “Being too embarrassed”. Individuals out of workforce with prolonged coughing were three times more likely to report “Being too embarrassed” and to report “Being worried about wasting the doctors time”. Individuals in the oldest age group and individuals out of workforce or on disability pension were less likely to report “Being too busy”.

### Implications

This study adds to the knowledge about barriers to healthcare-seeking with lung cancer symptoms in the general population and highlights several social inequalities. Individuals who currently smoke, immigrants and individuals out of workforce are in higher risk of postponing relevant healthcare-seeking due to barriers. Thus, future interventions and information should be targeted to aid these particularly vulnerable citizens and address their barriers. Information should be unprejudiced and emphasize that worrying about a symptom should lead to healthcare-seeking, not the opposite, and that evaluation of lung cancer symptoms is never a waste of the GP’s time. The overall strategies on how to reach the right individuals, and assure healthcare-seeking when indicated and relevant, may need some reconsideration in terms of more focus on interventions when e.g., the social workers in the communities meet the individuals to e.g., talk about their job situation. More individualised interventions may also be of great value to individuals with low health literacy in terms of e.g., lack of a social network or difficulties talking to the healthcare professionals. One suggestion could be a community-based mentor scheme, where the support is not limited to either healthcare- or community services but includes both^50^. A support system aimed to alleviate the challenges that may occur when individuals move from one sector to another, e.g., from primary healthcare to secondary healthcare or vice versa, may enhance the chance of individualized and coherent diagnostic paths for vulnerable patients. The current knowledge about the interplay between health literacy, socioeconomic status, smoking history, healthcare-seeking behaviour and how the latter can be supported by individualized initiatives is sparse and should be explored in future studies.

Further, knowledge about individuals who are at risk of postponing relevant healthcare-seeking with lung cancer symptoms is of utmost importance to the GPs who play a key role in the Danish healthcare system. By disseminating this knowledge the GP’s may be more aware of reaching out to vulnerable citizens. Thereby the likelihood of appropriate healthcare-seeking will be largely increased and the chances of timely diagnosis of lung cancer improved.

## Methods

### Study design and population

This study is part of a nationwide cohort study called the Danish Symptom Cohort (DaSC) designed to explore symptoms, healthcare-seeking and factors affecting healthcare-seeking behaviour in the general population. In 2012 a total of 100,000 individuals aged 20 years or older, randomly selected from the Danish Civil Registration System (CRS) were invited to participate in a survey. The selected individuals received an invitation by letter explaining the purpose of the study and containing a login to a secure web page. On the first page of the online questionnaire respondents consented to participation and agreed that their data could be used for research. Participants who could not access the online questionnaire were offered the option of completing over the telephone with a study member. Individuals, who had not responded within two weeks, received a new letter with a reminder, and those who had not responded after additional two weeks were contacted by telephone and encouraged to participate. Participation was voluntary, and all participants gave informed consent at the first page of the questionnaire. Participants stating not wishing to participate were excluded from the reminder procedure. Data was collected from June to December 2012. Individuals who were too ill, who migrated or died during the study period was excluded. Information on death and migration were obtained by register linkage, while information on severe illness was obtained by the invitees or their relatives contacting the project group and providing the information. Severe illness was primarily reported as dementia and terminal illness.

### The questionnaire

A questionnaire concerning experience of 44 symptoms with specific and non-specific cancer symptoms as well as frequent symptoms was developed. For each reported symptom additional questions regarding healthcare-seeking behaviour, and barriers to healthcare-seeking were asked. Symptoms of several cancers (lung, colorectal, gynaecological, and urological) were selected based on literature review including national and international cancer guidelines^51,52^. The questionnaire was based on standard rating scales, validated questionnaires, and ad hoc items. The methodological framework including details about the conceptual framework and the developing, pilot- and field tests is described in detail elsewhere^36^.

Participants were asked about symptom experiences, onset of the symptoms and healthcare-seeking behaviour, e.g., GP contact. For each symptom not presented to the GP questions concerning barriers to healthcare-seeking were asked. The included barriers originated from the Awareness and Beliefs About Cancer (ABC) measure^53^, which has been translated into Danish and validated in a Danish context^54^. The barriers were chosen based on the literature exploring anticipated barriers to healthcare-seeking^55^ and an already validated international measurement tool^53^. We included the following four predefined barriers in the study: “Being too embarrassed”, “Being worried about wasting the doctors time”, “Being worried about what the doctor might find” and “Being too busy”. Moreover, respondents were given the opportunity to describe other barriers in a free-text box. At the end of the questionnaire respondents were asked about lifestyle factors, such as smoking status. The phrasing of each question from the DaSC survey used in this study is presented in the Supplementary methods.

In the present study we included symptoms which might be signs of lung cancer. In the Danish lung cancer guideline specific lung cancer symptoms are defined as prolonged coughing (>4 weeks), shortness of breath, haemoptysis, and prolonged hoarseness (>4 weeks) among adults older than 40 years with a relevant smoking history. In addition, non-specific symptoms such as weight loss, loss of appetite and tiredness should raise suspicion of lung cancer, both alone and in combination with the specific lung cancer symptoms^56^. Based on the self-reported smoking status individuals were divided into three categories: individuals who never smoked, individuals who formerly smoked and individuals who currently smoke^57^.

### Register data

Socioeconomic data were obtained from statistics Denmark by using the individual identification numbers in the CRS^58–60^. Further, data on death or migration in the period of data collection was obtained. The data were obtained through register linkage, to keep the number of questions in the comprehensive survey low, and because the Danish registers are extensive and valid^58–60^. The variables of interest were highest obtained educational level, marital status, labour market affiliation, and ethnicity. Details of the variables are described under statistical analyses.

### Statistical analyses

According to the Danish guideline lung cancer should mainly be suspected in individuals older than 40 years, presenting with lung cancer symptoms, who have a current or former history of smoking^56^. Thus, we chose only to include respondents older than 40 years in the analyses. To be able to compare barriers towards healthcare-seeking between different risk groups, we both included individuals who never-, formerly-, and currently smoked in the study.

For each of the four specific lung cancer symptoms; prolonged coughing, shortness of breath, haemoptysis, and prolonged hoarseness, we used descriptive statistics to calculate the prevalence of the symptoms and the proportion of GP contacts. Furthermore, descriptive statistics was used to calculate the proportion of GP contacts with each specific lung cancer symptom, when reporting an additional specific lung cancer symptom, and when reporting both an additional specific and a non-specific lung cancer symptom (weight loss, loss of appetite and tiredness). These analyses were made to evaluate whether the combination of specific and non-specific lung cancer symptoms affected the healthcare-seeking behaviour.

Among individuals reporting each of the four specific lung cancer symptoms and no GP contact, we calculated the proportion of each of the four predefined barriers and the proportion of individuals reporting no barriers to healthcare-seeking by using descriptive statistics. Due to Danish legislation, reporting of the data for a number of individuals less than three is not permitted, thus results regarding barriers to healthcare-seeking with haemoptysis are not reported^61^.

For each of the symptoms; prolonged coughing, shortness of breath, and prolonged hoarseness we analysed the association between each barrier to healthcare-seeking and sex, age, smoking status, and socioeconomic factors, respectively. Multivariate logistic regression models were used to calculate both crude and adjusted odds ratios. The analyses were adjusted for age, smoking status, labour market affiliation, educational level, and ethnicity, which were the covariates showing significant associations with one or more barriers in the crude analyses. We calculated 95% confidence intervals using binomial distribution. Due to a small number of individuals reporting “Being too embarrassed” to contact the GP with prolonged hoarseness only crude analyses were made. We tested for interaction between sex, age, and smoking status. No interactions were found.

The covariates included in the analyses were categorized as follows: Age-groups: 40–54 years, 55–69 years, and 70 years or older. Smoking status: Individuals who never smoked, individuals who formerly smoked, and individuals who currently smoke. Highest obtained educational level: low (<10 years, i.e., primary/lower secondary school); middle (10–14 years, i.e., vocational or higher secondary school) or high (≥14 years, i.e. short-, medium-, or long-term higher education). Marital status: single or married/living together. Labour market affiliation: working, pension, out of workforce and disability pension. Ethnicity: Danish or immigrants/descendants of immigrants.

Data analyses were conducted using STATA statistical software 16.1 (StataCorp, College Station, TX, USA). All tests used a significance level of *p* < 0.05.

### Inclusion and ethics

The respondents were informed that participation in the study was voluntary. In the invitation letter thorough information about the purpose and content of the questionnaire was given. Respondents who had questions to the study had the opportunity to contact the project group by phone or email for clarification. The respondents were informed there would be no clinical follow-up and instructed to contact their doctor in case of concern. The Regional Scientific Ethics Committee for Southern Denmark was notified prior to the survey and found that no further approval was needed. The project has been approved by the Danish Data Protection Agency (j.no. 2011-41-6651) through the Research and Innovation Organisation (RIO), University of Southern Denmark (Project number 10.104).

### Reporting summary

Further information on research design is available in the Nature Research Reporting Summary linked to this article.

## Supplementary information

Supplementary Material

REPORTING SUMMARY

## Data Availability

The datasets generated and analysed in the current study are not publicly available and cannot be shared due to the data protection regulations of the Danish Data Protection Agency. Access to data is strictly limited to the researchers who have obtained permission for data processing. This permission was given to the Research Unit of General Practice, Department of Public Health, University of Southern Denmark. Further inquiries can be made to the PI Dorte Jarbøl email: DJarbol@health.sdu.dk

## Code availability

The statistical analyses were performed on the servers of Statistics Denmark and the coding is therefore not publicly available.
