# Supplementary material for: A population-based study on social inequality and barriers to healthcare-seeking with lung cancer symptoms
Source: NPJ Prim Care Respir Med. 2022 Nov 5;32:48. doi: 10.1038/s41533-022-00314-7 (PMC9637082; doi:10.1038/s41533-022-00314-7)
Supplement: Supplementary file 1 — Supplementary Material [file 41533_2022_314_MOESM1_ESM.pdf]

Supplementary methods: Questions from the Danish Symptom Cohort survey used in the study

**Variable(s)**	**Question(s)**	**Answer categories**
Symptom experience	*Have you within the last 4 weeks experienced any of these?*<br>*(You may tick more than one box)*	Coughing<br>Haemoptysis (coughing up blood)<br>Shortness of breath<br>Hoarseness<br>Weight loss > 2 kg<br>Tiredness<br>Loss of appetite
Additional questions asked for each reported symptom		
Onset	*When did you experience the symptom for the first time?*	< 4 weeks ago, 1-3 three months ago, 3-6 months ago and > 6 months ago
Contact to general practitioner	*Have you contacted your general practitioner with any of the following symptoms or discomforts? (Through appointment, by telephone or by email)*	Yes or no
Additional questions to the symptoms, which had not been presented to the general practitioner		
Barriers to healthcare-seeking	*You have **not** been in contact with your general practitioner regarding the following symptoms and discomforts. We would now like to know, whether you had some of the following considerations, regarding contact to your general practitioner?*<br>*(You may tick more than one box)*	I would be too embarrassed (yes/no)<br>I would be worried about wasting the doctor's time (yes/no)<br>I would be worried about what the doctor might find (yes/no)<br>I would be too busy to make time to go to the doctor (yes/no)<br>Other considerations (box for free text commentaries)
All respondents		
Smoking status	*Do you smoke?*	Yes, every day<br>Yes, at least once a week<br>Yes, less than once a week<br>No, I have stopped<br>No, I have never smoked
